# From WGS to gels: Development and testing of PCR primers targeting toxic *Digitalis* in support of food safety

**DOI:** 10.1002/aps3.70013

**Published:** 2025-07-01

**Authors:** Elizabeth Sage Hunter, Sydnee Fo, Robert Literman, Richard H. Uva, Jennifer L. Wolny, Sara M. Handy

**Affiliations:** ^1^ U.S. Food and Drug Administration, Center for Food Safety and Applied Nutrition Office of Regulatory Science 5001 Campus Dr. College Park 20740 Maryland USA; ^2^ U.S. Food and Drug Administration, Center for Food Safety and Applied Nutrition Office of Analytics and Outreach 5001 Campus Dr. College Park 20740 Maryland USA; ^3^ Seaberry Farm 2770 Wright Rd. Federalsburg 21632 Maryland USA; ^4^ Present address: U.S. Food and Drug Administration, Human Foods Program, Office of Laboratory Operations and Applied Science Office of Applied Microbiology and Technology 5001 Campus Dr. College Park 20740 Maryland USA; ^5^ Present address: U.S. Food and Drug Administration, Human Foods Program Office of Surveillance Strategy and Risk Prioritization 5001 Campus Dr. College Park 20740 Maryland USA

**Keywords:** dietary supplements, *Digitalis*, foods, foxglove, genomics, PCR, toxic plants

## Abstract

**Premise:**

This study capitalized on a library of single‐nucleotide polymorphisms created via whole genome sequencing (WGS) to develop and test a PCR assay for detecting toxic *Digitalis* species in food products. Complex foods can be difficult to analyze, but safeguarding consumer well‐being and public health necessitates that products regulated by the U.S. Food and Drug Administration are contaminant free.

**Methods:**

Ten pairs of PCR primers were designed, optimized, and tested against a subset of vouchered specimens. Two primer sets were screened using 55 vouchered Plantaginaceae species, complex food matrices, five different plant tissues, a dilution series, and spiked food products with 0.5%, 1%, and 5% biomass of *D. purpurea* and *D. lanata*.

**Results:**

At optimized annealing temperatures, these primers amplified only *Digitalis* spp. (5). Both primer sets could detect spikes of *D. purpurea* and *D. lanata* down to 0.5% biomass and across three orders of magnitude, as well as five tissue types of *D*. *purpurea*.

**Discussion:**

This study provides an enhanced DNA‐based method for detecting *Digitalis* in complex food products. This novel method of primer development from WGS data lays the groundwork for a larger, more comprehensive panel for the rapid identification of botanical contaminants that may pose risks to consumers.

Numerous plants have evolved to produce toxic compounds as a means of defense against herbivores and predation, microorganisms, carbon cycling, and environmental conditions (Bryant et al., 1983; Kocyigit et al., 2023). Phytotoxins, such as abrin (*Abrus precatorius* L., rosary pea), ricin (*Ricinus communis* L., castor bean), and aconitine (*Aconitum* L., monkshood), are extremely potent, with a few leaves or seeds capable of causing human fatalities without prompt treatment (Worbs et al., 2011; Karthikeyan and Amalnath, 2017; San Andrés Larrea et al., 2024). While many phytotoxins are not quite this dangerous, any introduction of toxic species into the food supply can cause adverse health effects, and the rapid detection of species of concern is critical. Due to the globalized supply chain for bulk botanical materials, a finished product may contain ingredients harvested all over the world and sourced from dozens of different distributors. These ingredients are often milled, processed, extracted, or otherwise devoid of morphological characters by the time they reach the manufacturer, and more so by the time they reach the consumer. Therefore, DNA‐based identification methods are needed to ensure that consumer products and their precursors are free from toxic species. These methods should ideally be rapid, practical, and scalable so that both raw materials and end products can be screened simultaneously, and the sources of potential botanical contamination promptly identified.

Incidents of adulteration of food products and dietary supplements can arise due to intentional, economically motivated adulteration (Khilare et al., 2019); misidentification or accidental harvest (Slifman et al., 1998; Maffè et al., 2009; Worbs et al., 2011; Walker and Applequist, 2012; Boskabadi et al., 2021; Mutebi et al., 2022; Gafner et al., 2023); or the inclusion of novel ingredients not generally recognized as safe (GRAS) by the U.S. Food and Drug Administration (FDA) (Chan and Smith, 2023). These issues can also extend to animal feed (Worbs et al., 2011; San Andrés Larrea et al., 2024). Contamination with toxic plant species most often occurs accidentally; incidents have arisen from individuals foraging, buying mislabeled products from boutique markets, or when toxic congeners or look‐alikes are mistakenly harvested alongside edible species (Slifman et al., 1998). In this study, we specifically target the toxic genus *Digitalis* L., building on previous research by Hunter et al. (2021) that described the use of whole genome sequencing (WGS) data to detect the presence of this taxon in in silico mockups of dietary supplements.

*Digitalis* is a small genus of about 23 (Kreis, 2017) to 35 (World Flora Online, 2024) species in the family Plantaginaceae and is commonly known as foxglove. All but one species in this genus has been found to produce cardiac glycosides, specifically cardenolides, which act on the heart (Kreis, 2017). These plants are popular ornamental species and, as a result of human cultivation, are now naturalized across much of North America in addition to their large native range in Europe, Asia, and North Africa (Kreis, 2017). While *Digitalis* has a long history of medicinal use dating back to pharmaceutical work by William Withering in the late 1700s, the therapeutic index for the cardiac glycosides it produces is extremely narrow, and it is known to cause a number of severe effects when therapeutic levels are exceeded and toxicity ensues (Cushny, 1925; Beller et al., 1971). These effects include gastrointestinal symptoms, hyperkalemia, hallucinations, delirium, weakness, fatigue, and severe cardiac manifestations which can be lethal (Lapostolle and Borron, 2007; Pincus, 2016). *Digitalis* has contaminated both dietary supplements, when it was accidentally harvested alongside *Plantago* L. (Slifman et al., 1998), and food products, when it was mistaken for the leaves of the edible species *Borago officinalis* L. (Maffè et al., 2009; Negroni et al., 2019), *Symphytum officinale* L. (Lin et al., 2010), and *Plantago lanceolata* L. (Castello et al., 2012).

Reliably determining the botanical elements of complex matrices, such as dietary supplements or food products, presents a significant methodological challenge. These products are extremely varied in composition, complexity, level of processing, fat and salt content, and physical state. Because the residual DNA in highly processed products can be fragmented and present in overall low concentrations, it is important to have controls in place to account for these obstacles. Despite these challenges, a variety of DNA‐based identification methods have been used to authenticate food products, and DNA barcoding/metabarcoding as a means of identification has been widely explored. However, universally conserved priming targets in vertebrates, like those the FDA uses for seafood identification (Handy et al., 2011), are typically not discerning enough to be used independently in plants (*matK*, *rbcL*, *trnH‐psbA*, and ITS), so they are often used in concert to improve taxonomic identification (Li et al., 2015). Metabarcoding a suite of target loci in a mixed botanical product requires next‐generation sequencing and subsequent complex downstream bioinformatic analyses to determine mixture components (Coutinho Moraes et al., 2015; Ivanova et al., 2016). Genome skimming and the use of chloroplast genomes as a “super barcode” have also been explored to overcome the limitations of barcoding and metabarcoding (Li et al., 2015; Zhang et al., 2017). While these methods have value as untargeted, holistic approaches, they rely on large, curated databases, struggle with highly degraded or trace quantities of DNA, and are time consuming to perform (Coutinho Moraes et al., 2015; Ivanova et al., 2016; Breitwieser et al., 2019; Bohmann et al., 2020; Pearman et al., 2020). In emergent situations, where human health is at risk, fast assays for specific species of concern, especially those that use simplified equipment and inexpensive reagents, are valuable tools.

Hunter et al. (2021) leveraged a modified implementation of Site Identification from Short Read Sequences (SISRS; Schwartz et al., 2015) to identify *Digitalis* in simulated spiked genome skimming data based on the case report described in Slifman et al. (1998). The SISRS method is a reference‐free ortholog discovery pipeline, and its application is particularly advantageous in taxa where reference genomes are limited, including many toxin‐producing plant species of potential concern for the FDA. As of 2017, less than 1% of angiosperms were fully sequenced (Chen et al., 2018), and as of 2024, GenBank RefSeq still contains less than 1000 chromosome‐level angiosperm assemblies (accessed 15 July 2024). Hunter et al. (2021) identified over 2 million informative genus‐level single‐nucleotide polymorphisms (SNPs) and thousands of species‐specific SNPs that could accurately distinguish *Digitalis* from closely related species in the Plantaginaceae and among species within the genus in simulated samples. Building on these compelling results, the existing SISRS dataset was mined to develop a PCR assay to rapidly screen for and identify *Digitalis* contamination in complex botanical food mixtures, making identification of potential adulterants faster and more viable for a variety of users.

## METHODS

### Primer design

Sequence data from Hunter et al. (2021) and publicly available sequence data spanning seven genera in the family Plantaginaceae (*Digitalis*, *Plantago*, *Veronica* L., *Callitriche* L., *Bacopa* Aubl., *Littorella* P.J. Bergius, and *Gratiola* L. for a total of 32 individuals) were utilized to develop a suite of 10 primer pairs intended to be specific for *Digitalis* (Table 1). This was conducted using the existing pangenome SNP dataset from Hunter et al. (2021), generated using the SISRS pipeline (Schwartz et al., 2015). This pipeline evenly sampled WGS data from representatives of the *Digitalis* genus, pooled it into a mixed species dataset, and assembled it together to generate a single composite pangenome assembly. The data were then filtered by mapping datasets from closely related species in the family Plantaginaceae, as well as the genus *Digitalis*, onto the pangenome assembly to identify sites unique to the desired taxa at both the genus and species level. Any sites with homozygous coverage and sufficient read depth were called as fixed sites. All sites that were fixed at either the genus or species level were identified as potential targets for primer design. To increase *Digitalis* specificity, these data were filtered by removing any sites with read coverage in non‐*Digitalis* Plantaginaceae species, resulting in 2.4 million *Digitalis*‐specific SNPs and species‐specific *Digitalis* SNP datasets ranging from 6.6 to 101 kbp.

**Table 1:** Novel nuclear primers (RAL01–RAL10) for *Digitalis* identification developed and tested for this study, including primer sequences, predicted amplicon length based on the SISRS composite genome, and the number of species‐informative SNPs predicted per amplicon. Selected primers RAL08 and RAL10 are shown in bold.

Primer name		Amplicon length (bp)	No. of SNPs predicted per amplicon				
	Primer sequences (5′–3′)		*D. ferruginea*	*D. grandiflora*	*D. lanata*	*D. lutea*	*D. purpurea*
RAL01 F	AATGTTGCTGCAATCTGCA	802	2	5	3	2	7
RAL01 R	GAAGCGCATTGTTGAAGC						
RAL02 F	CATGAGCTGAGGACAATGAT	839	2	4	1	1	25
RAL02 R	GATACTATAACAGAGCCGCTG						
RAL03 F	TCCATCGCATCTAGAACTTC	352	1	2	0	3	13
RAL03 R	ATTCATTGCAGCAAAACCAA						
RAL04 F	AAGAGTGAGAAAGGAACAA	625	2	4	2	1	5
RAL04 R	TCAAAAGGATATGAGTTTGTTA						
RAL05 F	GATTAGGACATACTTCAGCAGAG	922	2	1	3	3	31
RAL05 R	GACAATTTTGCCTAGATGAAATTAG						
RAL06 F	CTATTTCAATCCTAGCTCGTCA	823	2	3	2	1	13
RAL06 R	TCTAGTGCTTCTGTGGCTAA						
RAL07 F	TTGCATTCCAATTGCATAG	844	1	0	3	1	7
RAL07 R	ATGATTGCTCTGGATTCTGC						
**RAL08 F**	**AGGCTTTTCTCGCTTAGTATC**	**688**	**11**	**1**	**3**	**2**	**8**
**RAL08 R**	**CCTTGCCTCTAAACTTTATCTTC**						
RAL09 F	TTGGTTCGTAGACTGAAAGC	224	0	0	2	0	9
RAL09 R	GAGGTTGAACAGCCAAGTC						
**RAL10 F**	**GTGTTCCTAGGATATGGTTTTGT**	**211**	**2**	**2**	**1**	**0**	**5**
**RAL10 R**	**AAGTGGCATGCAGTTGATT**						

To identify genomic regions where presence/absence PCR primers for *Digitalis* detection could be used, the datasets were first assessed with BEDTools (Quinlan and Hall, 2010) to identify 25‐bp windows with high densities of genus‐specific SNPs. From these, paired regions that were a suitable distance apart for PCR were identified and BEDTools was used to tally the number of species‐specific SNPs in the interval. This allowed for the identification of 86 intervals on 69 contigs where dense *Digitalis‐*specific windows were ~200–1000 bp apart and contained species‐specific SNPs. These data were loaded into Geneious Prime (version 2023.1; https://www.geneious.com), and using different colors for each species, regions with high SNP variance were visually identified. Of the 86 computationally identified regions, 10 were compatible with primer design in areas surrounding high numbers of species‐specific SNPs (Table 1). These 10 primer pairs were purchased from Integrated DNA Technologies (Coralville, Iowa, USA) and diluted to a working stock of 10 µM when received.

### Voucher collection and extraction

**Figure 1:**
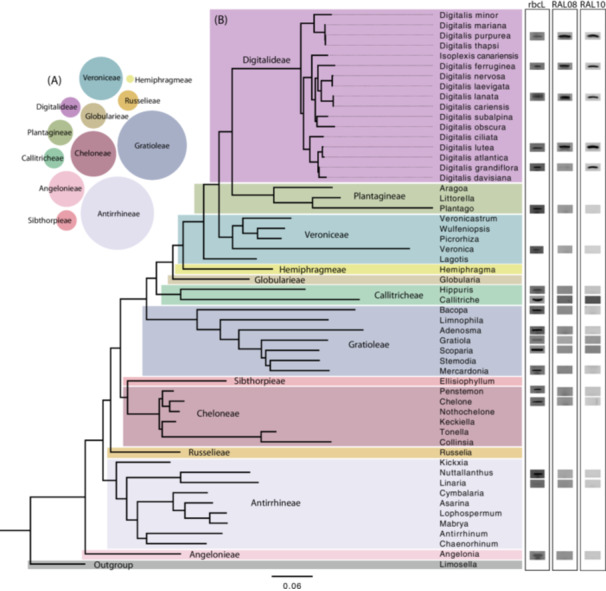
Relative size and phylogenetic distribution of taxa used to validate *Digitalis* primers designed for this study. (A) The relative sizes of the 12 tribes of Plantaginaceae represented in a normalized bubble plot based on the number of genera in each tribe. (B) FastTree ITS2 gene tree showing the 12 tribes of Plantaginaceae and a band obtained for each genus using the *rbcL* control primers, and the RAL10 and RAL08 primers designed and validated in this study.

Sixty‐two individuals representing 15 genera and 46 species across a broad range of the Plantaginaceae family (Figure 1) were obtained from the New York Botanical Garden (NYBG), Seaberry Farm, the National Center for Natural Products Research (NCNPR), the Smithsonian Institution's National Museum of Natural History (Botany Department), University of Rhode Island's Heber W. Youngken Medicinal Garden (URI), and Green Farmacy Garden. One sample was collected in Placita, Colorado, USA, by one of the authors (J.L.W.) (Table 2). These included a total of 12 representatives of *Digitalis*, including one each of *D. ferruginea* L., *D. grandiflora* Mill., and *D. lutea* L.; two of *D. lanata* Ehrh.; and seven of *D. purpurea* L. The *D. purpurea* representatives included six cultivars: ‘Camelot Cream’, ‘Camelot Lavender’, ‘Alba’, ‘Lavender Carousel’, ‘Primrose Carousel’, and ‘Apricot Beauty’.

**Table 2:** List of the voucher specimens used as part of this study, including their identifier, scientific name, source, and columns indicating positive or negative PCR results (band or no band) for the *rbcL* primer set, RAL08 primer set, and RAL10 primer set.

ID	Species	Source	*rbcL*	RAL08	RAL10
NYBG_2761026	*Adenosma glutinosa* var. *caerulea* (R.Br.) Tsoong	NYBG	+	−	−
NYBG_1283229	*Angelonia angustifolia* Benth.	NYBG	+	−	−
NYBG_1284644	*Bacopa monnieri* (L.) Wettst.	NYBG	+	−	−
NYBG_2756319	*Bacopa monnieri* (L.) Wettst.	NYBG	+	−	−
NYBG_1125745	*Callitriche heterophylla* Pursh	NYBG	+	−	−
NYBG_1120822	*Callitriche stagnalis* Scop.	NYBG	−	NA	NA
NYBG_1283494	*Chelone glabra* L.	NYBG	+	−	−
NYBG_1120544	*Digitalis ferruginea* L.	NYBG	+	+	+
NYBG_1124823	*Digitalis ferruginea* L.	NYBG	−	NA	NA
NCNPR #9899	*Digitalis ferruginea* L.	NCNPR	+	+	+
NYBG_1120780	*Digitalis ferruginea* L.	NYBG	−	NA	NA
NYBG_1120013	*Digitalis grandiflora* Mill.	NYBG	+	+	+
GF_20	*Digitalis lanata* Ehrh.	Green Farmacy Garden	+	+	+
NCNPR #23694	*Digitalis lanata* Ehrh.	NCNPR	+	+	+
URI10	*Digitalis lutea* L.	URI Medicinal Garden	+	+	+
NCNPR #1603	*Digitalis purpurea* L.	NCNPR	+	+	+
Camelot Cream‐1	*Digitalis purpurea* L.	Seaberry Farm	+	+	+
Camelot Lavender‐1	*Digitalis purpurea* L.	Seaberry Farm	+	+	+
Digitalis Alba‐1	*Digitalis purpurea* L.	Seaberry Farm	+	+	+
Lavender Carousel‐1	*Digitalis purpurea* L.	Seaberry Farm	+	+	+
Primrose Carousel‐1	*Digitalis purpurea* L.	Seaberry Farm	+	+	+
Apricot Beauty‐1	*Digitalis purpurea* L.	Seaberry Farm	+	+	+
NYBG_1121451	*Gratiola ramosa* Walter	NYBG	+	−	−
Wen 17756	*Hippuris vulgaris* L.	Smithsonian	+	−	−
NYBG_1120228	*Linaria canadensis* (L.) Dum.Cours.	NYBG	+	−	−
NYBG_2757124	*Linaria vulgaris* Mill.	NYBG	+	−	−
Wen 17313	*Mecardonia acuminata* (Walter) Small	Smithsonian	+	−	−
NYBG_1125042	*Mecardonia acuminata* (Walter) Small	NYBG	+	−	−
NYBG_2758543	*Nuttallanthus canadensis* (L.) D.A. Sutton	NYBG	+	−	−
NYBG_1122553	*Penstemon digitalis* Nutt. ex Sims	NYBG	+	−	−
NYBG_1122550	*Penstemon digitalis* Nutt. ex Sims	NYBG	+	−	−
NYBG_2758238	*Plantago arenaria* L.	NYBG	+	−	−
NYBG_1281969	*Plantago aristata* Michx.	NYBG	+	−	−
NYBG_1276287	*Plantago erecta* E. Morris	NYBG	+	−	−
NYBG_1283601	*Plantago helleri* Small	NYBG	−	NA	NA
NYBG_1281903	*Plantago lanceolata* L.	NYBG	+	−	−
NYBG_1122903	*Plantago lanceolata* L.	NYBG	+	−	−
NYBG_2757108	*Plantago major* L.	NYBG	+	−	−
NYBG_2930491	*Plantago major* L.	NYBG	+	−	−
NYBG_1125344	*Plantago maritima* L.	NYBG	+	−	−
NYBG_1120571	*Plantago media* L.	NYBG	−	NA	NA
NYBG_1283128	*Plantago patagonica* Jacq.	NYBG	+	−	−
NYBG_1283665	*Plantago rhodosperma* Decne.	NYBG	−	NA	NA
Wen 12971	*Plantago rugelii* Decne.	Smithsonian	+	−	−
NYBG_1125906	*Plantago rugelii* Decne.	NYBG	+	−	−
NYBG_1282012	*Plantago virginica* L.	NYBG	+	−	−
NYBG_2756357	*Scoparia dulcis* L.	NYBG	+	−	−
NYBG_1281521	*Veronica anagallis‐aquatica* L.	NYBG	+	−	−
NYBG_2758066	*Veronica arvensis* L.	NYBG	+	−	−
NYBG_1282507	*Veronica chamaedrys* L.	NYBG	+	−	−
NYBG_1124646	*Veronica gentianoides* Vahl	NYBG	+	−	−
NYBG_2758305	*Veronica hederifolia* L.	NYBG	+	−	−
NYBG_1120762	*Veronica liwanensis* K. Koch	NYBG	−	NA	NA
NYBG_1120580	*Veronica nigricans* K. Koch	NYBG	−	NA	NA
NYBG_2759029	*Veronica officinalis* L.	NYBG	+	−	−
NYBG_2758064	*Veronica peregrina* var. *peregrina* det. D.E. Atha	NYBG	+	−	−
NYBG_2758183	*Veronica persica* Poir.	NYBG	+	−	−
NYBG_1282047	*Veronica polita* Fr.	NYBG	+	−	−
NYBG_2757191	*Veronica scutellata* L.	NYBG	+	−	−
NYBG_2757982	*Veronica serpyllifolia* var. *serpyllifolia* det. D.E. Atha	NYBG	+	−	−
NYBG_1282819	*Veronica spicata* L.	NYBG	+	−	−
NYBG_1120464	*Veronica telephiifolia* Vahl	NYBG	+	−	−
Wolny2024_PS	*Penstemon strictus* Benth.	Placita, Colorado, USA	+	−	−

*Note*: NA, not applicable; NCNPR, National Center for Natural Products Research; NYBG, New York Botanical Garden.

Leaves and flowers from the fresh cultivars of *D. purpurea* collected from Seaberry Farm were dried using a large‐scale plant press comprising two wooden pallets, several layers of cardboard and paper, and ratchet straps. Additional weight was added to the top of the press to provide sufficient pressure, and a fan was run in the vicinity to ensure adequate ventilation of the fully loaded press. Individual plant specimens were evenly spaced and marked on each layer. Samples were pressed for approximately eight weeks and then bagged and stored at 4°C on silica gel. Similar methods were employed to dry the *Penstemon strictus* Benth. material collected in Colorado. The plant material from NYBG, NCNPR, URI, and the Smithsonian arrived dried and ready for use; these samples were stored at −20°C until used.

DNA was extracted from the 62 voucher samples using a Qiagen DNeasy Plant Mini Kit (Qiagen, Germantown, Maryland, USA) and quantified on an Invitrogen Qubit fluorometer (Invitrogen, Waltham, Massachusetts, USA).

### Primer selection and specificity

For all PCR assays, the Invitrogen Platinum *Taq* DNA Polymerase kit was used along with trehalose as described in Handy et al. (2011). Each reaction contained 6.25 μL of 10% trehalose solution, 2 μL of double‐distilled H_2_O, 1.25 μL of 10× PCR buffer, 0.625 μL of 50 mM MgCl_2_, 0.125 μL of 10 μM of both primers, 0.062 μL of 10 mM dNTPs, 0.060 μL of Platinum *Taq* (5 U/μL), and 1 μL of undiluted DNA template (unless noted) per reaction, for a total volume of 11.5 μL. In all sets of reactions, positive controls and both extraction and PCR negative controls were used. An Eppendorf Mastercycler ep gradient S thermocycler (Eppendorf North America, Enfield, Connecticut, USA) or a Bio‐Rad T100 Thermal Cycler (Bio‐Rad, Hercules, California, USA) was used for each PCR. A general *rbcL* primer set (rbcLaF/rbcLaR; Fazekas et al., 2008) or general ITS2 primer set (S2F/S3R; Chen et al., 2010) was used to ensure the DNA samples contained uninhibited, amplifiable DNA. Thermocycler conditions used for these primer sets are detailed in Table 3. The *Digitalis‐*specific primer sets were all run on the same basic set of cycles with a range of annealing temperatures determined by the gradient tests described below. Products were verified using Invitrogen precast 2% E‐gel agarose gels according to the manufacturer's protocols with the E‐Base Integrated power supply. Gels were run for 5–15 min and then visualized using a Syngene G:BOX Chemi XRQ imaging system (Syngene USA, Iselin, New Jersey, USA).

**Table 3:** Final primer cycles for the control targets *rbcL* (rbcLaF/rbcLaR) and ITS2 (S2F/S3R), as well as the general cycle and range of annealing temperatures (*) used to test all RAL primers in this study and the specific cycles optimized for the chosen primer sets RAL08 and RAL10.

Name	Initial denaturing	Denaturing and annealing cycle	Final extension	Hold	Source
rbcLaF/rbcLaR	96°C/3 min	(94°C/1 min 50°C/1 min 72°C/1 min) × 35	72°C/5 min	12°C	Fazekas et al. (2008)
S2F/S3R	94°C/5 min	(94°C/30 s 56°C/30 s 72°C/45 s) × 40	72°C/10 min	12°C	Chen et al. (2010)
General RAL F/RAL R	95°C/3 min	(95°C/30 s *58–60°C/30 s 72°C/1 min) × 35	72°C/5 min	12°C	This study
RAL10 F/RAL10 R	95°C/3 min	(95°C/30 s 56°C/30 s 72°C/1 m) × 35	72°C/5 min	12°C	This study
RAL08 F/RAL08 R	95°C/3 min	(95°C/30 s 60°C/30 s 72°C/1 min) × 35	72°C/5 min	12°C	This study

Before running the larger sample sets, an annealing temperature gradient was run on all primer pairs to determine ideal annealing temperatures. In this set of reactions, the extracted DNA of *D. purpurea* ‘Alba’ was used as a template and the range of annealing temperatures tested ranged from 50–60°C; a subsequent gradient was conducted with *D. purpurea* ‘Camelot Lavender’. Next, a subset of individuals that included up to three *Digitalis* species and two other non‐*Digitalis* Plantaginaceae species were tested to examine primer pair performance.

Successfully extracted members of the Plantaginaceae family were first screened for amplifiable DNA using *rbcL* primers as described above. Following this, all remaining individuals from the Plantaginaceae family were tested for specificity using the selected primer sets at their optimal annealing temperatures (Table 3).

In addition to testing these closely related individuals, the primers were also evaluated for specificity using 13 mixed botanical consumer products including spices and teas representing a wide array of taxa (Appendix S1). These consumer products were extracted using a Qiagen DNeasy Plant Mini Kit and quantified on an Invitrogen Qubit fluorometer. The full list of ingredients included 69 botanically derived, non‐Plantaginaceae ingredients. These 13 samples were also amplified with the general *rbcL* primer set used with the voucher samples to confirm PCR viability. A blend of two of these products (EM‐27 and EM‐28) was also used in the spiking experiments described below.

### Clean up and sequencing of RAL08 amplicons

While the PCR assay was designed to be genus specific, the primer set RAL08 was additionally designed to allow for species‐level identification with sequencing, if desired. While this was the intention of the design of the primer set using the WGS data (Table 1), it needed to be tested by bidirectionally sequencing the RAL08 amplicons generated from *Digitalis* species vouchers. To accomplish this, the PCR products from the *Digitalis* vouchers were cleaned by adding 2 μL of ExoSAP‐IT (Thermo Fisher Scientific, Waltham, Massachusetts, USA) to 5 μL of PCR product and incubating on a thermocycler at 37°C for 15 min, followed by 15 min at 80°C. Subsequently, the cleaned products were added to a sequencing reaction in the following mix: 0.25 μL of BigDye Terminator v3.1 (Thermo Fisher Scientific); 1.875 μL of 5× sequencing buffer; 5 μL of 10% trehalose; 1 μL of 10 μM primer (either RAL08F or RAL08R); and 0.875 μL of molecular‐grade water, for a total of 9 μL, and 1 μL of cleaned‐up PCR product. At a minimum, one representative of each species was selected, and sequencing reaction products were purified as illustrated in Handy et al. (2011) using the EdgeBio Performa DTR short‐well plate kit (Edge Bio Systems, Gaithersburg, Maryland, USA). Purified samples were placed on a Sanger ABI 3500 instrument (Applied Biosystems, Waltham, Massachusetts, USA) and bidirectionally sequenced.

**Figure 2:**
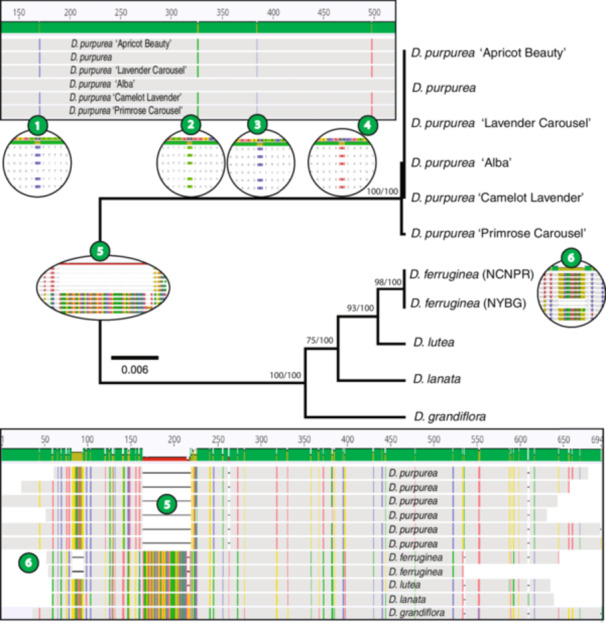
*Digitalis* RAL08 UPGMA tree with overlaid RAxML support values. The UPGMA bootstraps are shown on the left and the RAxML bootstraps are on the right. The alignment used to generate the tree is shown below, with windows 1–4 showing the four degenerate bases present in *D. purpurea* ‘Alba’ in the alignment of all the *D. purpurea* cultivars tested. Window 5 shows the 55‐bp deletion specific to the *D. purpurea* branch of this tree and window 6 shows the 13‐bp deletion specific to *D. ferruginea*.

The sequences were processed using Geneious Prime version 2023.1. First, reads were combined into contigs that were trimmed and edited manually to remove primer sequences and ambiguous bases at the sequence ends, then aligned using the MUSCLE plugin for Geneious Prime (version 5.1; http://www.drive5.com/muscle/), and phylogenetic trees were generated using the unweighted pair group method with arithmetic mean (UPGMA) in RAxML (Stamatakis, 2014) (Figure 2). The UPGMA tree was bootstrapped 1000 times. RAxML was run with the GTR+GAMMA model using rapid bootstrapping parameters (‐f a, ‐x) and 100 distinct starting trees (‐N 100).

### Ruggedness testing

A series of ruggedness experiments was designed to test the abilities and limitations of the assay with the chosen primer sets. Various tissues including flower, stem, seed pod, leaf, and roots of *D. purpurea* (two cultivars, ‘Alba’ and ‘Camelot Lavender’) were tested to discern any variation in extraction efficiency or quality of the DNA template. In addition to the specificity testing described above, the primers were tested with two species of *Digitalis* (*D. lanata* and *D. purpurea*) via a spike experiment and a serial dilution experiment to illustrate both assay sensitivity and the limit of detection.

#### Tissue tests

To confirm that the extraction protocol and primers worked on all tissue types, previously extracted leaf tissues from *D. purpurea* ‘Camelot Lavender’ and *D. purpurea* ‘Alba’ were used as positive controls for this experiment, which also included DNA extractions from stems, seed pods, flowers, and roots from the same two cultivars. These tissues were extracted using the Qiagen DNeasy Plant Mini Kit, quantified with a Qubit fluorometer, and amplified with RAL08 and RAL10 in a PCR as described above.

#### Leaf spikes and sensitivity testing

To test the sensitivity of the PCR primers in commercial products, leaf tissue of *D. purpurea* ‘Camelot Lavender’ (ground using an IKA Tube Mill 100 [IKA Works, Wilmington, North Carolina, USA] at 5000 rpm for 1.5 min) and *D. lanata* (previously ground at NCNPR) were spiked into a blended food sample matrix made from a mixture of two of the 13 commercial products (EM‐27 and EM‐28) previously used to test for primer specificity (Appendix S1). The listed ingredients in this matrix blend of commercial products included: marjoram, basil, rosemary, thyme, oregano, savory, sage, onion, garlic, black pepper, red bell pepper, tomato, corn derivatives, sunflower oil, vinegar, parsley, and turmeric extract. These products were blended using an IKA Tube Mill 100 until finely ground (5000 rpm for 35 s for two cycles in two IKA vessels), combined, and rotated at 30 rpm for 1 h to mix. The species for the *Digitalis* spikes were chosen because large amounts of tissue were available and because these species were noted in the literature to have contaminated foods and dietary supplements. Spikes were made by combining 6.5 g of the commercial product matrix with 0%, 0.5%, 1%, and 5% biomass by dry weight of each species (separately). Duplicate samples of 200 mg were extracted on two different days from each mix, using the Qiagen DNeasy Plant Mini Kit on the first day and the Qiagen Mericon Food Kit on the second day. Extracted DNA was analyzed using the same three PCR approaches as described above with the ITS2 primer set used as the control assay and *D. purpurea* ‘Camelot Lavender’ leaf material used as the positive control.

#### Limit of detection

The limit of detection (LOD) was assessed using a dilution series with the same two individuals used for the spike experiment, *D. purpurea* ‘Camelot Lavender’ and *D. lanata*. The DNA from both species was diluted in elution buffer in a 10‐fold series starting with a concentration of 10 ng/μL. Six dilutions were made and consequently the lowest level tested was 0.001 ng/μL. The PCRs using RAL08 and RAL10 were conducted as described above (1 µL was added of each template, for 10 ng, 1 ng, 0.1 ng, 0.01 ng, 0.001 ng, and 0.0001 ng per PCR, respectively).

### Figure generation

For Figure 1, a total of 1823 ITS2 sequences in the family Plantaginaceae were retrieved from the National Center for Biotechnology Information (NCBI) GenBank database using the built‐in search tools, with 16 of these representing *Digitalis*. These sequences were collapsed at 97% sequence similarity using CD‐HIT (Fu et al., 2012) and then aligned with MAFFT version 7.305b (Katoh and Standley, 2013) using the auto flag. The alignment was trimmed to 998 bp to remove overhang and then manually curated to remove erroneous and low‐quality sequences in Geneious Prime version 2023.1. All *Digitalis* sequences were added in after collapsing with CD‐HIT to ensure the genus was represented in sufficient detail. Similarly, it was ensured that all genera tested in this study were represented in the final alignment. Finally, the tree was constructed with FastTree version 2.1.11 (Price et al., 2010). This process was completed iteratively until a suitable tree was constructed. The GenBank accession numbers for the 334 sequences used in the final tree are available in Appendix S2.

The bubble plot in Figure 1A was constructed in R version 4.4.1 (R Core Team, 2024) using ggplot2 (Wickham, 2016) with data from table 1 in Albach et al. (2005). Each bubble is scaled to represent the relative size of each the 12 tribes of Plantaginaceae, using the number of genera each tribe contains. These counts were normalized by dividing by the largest number (28, Antirrhineae) and multiplying by a factor of 10.

## RESULTS

### Voucher DNA extraction

Of the vouchers, 55 specimens were found to have usable DNA via amplification with a general primer set (*rbcL*; Fazekas et al., 2008), including 13 representatives of *Digitalis* (two of the *D. ferruginea* failed to extract; Table 2). Amplifiable DNA concentrations ranged from less than 0.2 ng/µL (out of range for Qubit, which was the case for two specimens) to 55.3 ng/µL. A subset of these was used to test the 10 pairs of primers (RAL01–RAL10).

### Primer selection and specificity

Examination of the gradient gel revealed that the ideal annealing temperatures ranged from 56–60°C, with primer sets RAL01, 02, 04, 05, 06, 08 at 60°C, RAL07 at 58°C, and RAL09 at 59°C. RAL03 was faint at all temperatures. Primer set RAL10 was initially set at 60°C; however, an adjustment was made for reasons described below and in the Discussion that moved the annealing temperature to 56°C in the final assay (Appendix S3).

Experimentation with primer sets RAL03 and RAL09 was discontinued after they failed to amplify *D. purpurea* ‘Alba’ effectively. The remaining primer sets exclusively amplified all *Digitalis* species and no other species of Plantaginaceae in the initial subsets. Two primer sets (RAL08 and RAL10) were chosen to perform additional expansive testing (Table 1). These sets were chosen because they produced bright bands for the *Digitalis* species tested, showed no off‐target amplification in the initial test series, and because they each offered an additional benefit. RAL08 amplifies a longer insert (688 bp) that is species specific if sequenced, while RAL10 targets approximately 200 bp, which is beneficial for samples that have a high proportion of fragmented DNA, as might be encountered with processed food or dietary supplement products.

Initially, many cultivars of *D. purpurea* were not amplifying with primer set RAL10 with a 60°C annealing temperature. To further investigate this issue, an additional gradient PCR was conducted with cultivar *D. purpurea* ‘Camelot Lavender’ to reconfirm the optimal annealing temperature. At 50–55°C, all *Digitalis* individuals but also non‐*Digitalis* members of Plantaginaceae were amplified (Appendix S4), but an annealing temperature of 56°C amplified all *Digitalis*, including all cultivars of *D. purpurea*, and excluded all other members of Plantaginaceae.

Finally, the primers were tested against the full set of 55 Plantaginaceae species, including all available *Digitalis* species. Only *Digitalis* species amplified with both primer sets (Figure 1, Table 2). This was further confirmed using the set of 13 mixed botanical commercial samples with 69 non‐Plantaginaceae botanical ingredients listed on the label. The commercial samples were also tested with both RAL08 and RAL10 at their respective optimized annealing temperature to screen for off‐target amplification beyond Plantaginaceae. In these mixed samples, all controls functioned as expected. The general *rbcL* primer set indicated there was amplifiable DNA present, but no background amplification was detected for RAL08 or RAL10. Taken together, this verified the specificity of the primer sets.

### Species identification

The RAL08 amplicon alignment was a total of 778 bp in length and was divergent enough that all five *Digitalis* species tested could be distinguished with this marker. The *D. purpurea* cultivars were virtually identical: the cultivars ‘Lavender Carousel’, ‘Camelot Lavender’, and ‘Apricot Beauty’ were identical to each other (100%) and ‘Primrose Carousel’ had a 2‐bp difference (99.8% identical). All *D. purpurea* cultivars possessed a unique 55‐bp indel in this region, and *D. ferruginea* L. also had a characteristic 13‐bp indel (Figure 2). While the differences between *D. lutea*, *D. lanata*, and *D. grandiflora* were slightly more subtle, these species were also easily distinguished with nucleotide substitutions (Figure 2). Within this set, *D. lutea* and *D. grandiflora* were the most divergent with 16‐bp differences, while *D. lanata* and *D. lutea* were the most similar with 13‐bp differences.

The *D. purpurea* cultivar ‘Alba’, while matching the cultivars ‘Lavender Carousel’, ‘Camelot Lavender’, and ‘Apricot Beauty’ in all other alignment positions, contained polymorphic bases in four locations, which was different from all other *Digitalis* species tested (Figure 2). One was an R (A or G) at position 553, whereas all other cultivars and species of *Digitalis* had an A at this location. Additionally, at position 382 there was a Y (C or T) where the others had a T, and at positions 225 and 440 there was a Y where the others had a C (Figure 2).

The GenBank accessions for barcodes from all species and cultivars sequenced as part of this study are listed in Appendix S2.

### Tissue tests

**Figure 3:**
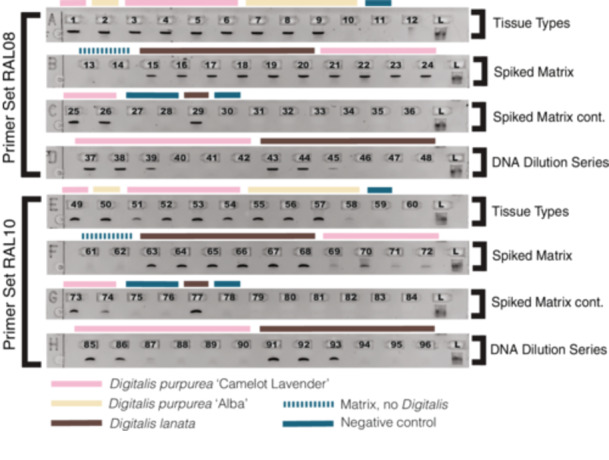
Gel from spike and serial dilution experiments. Rows A–D show primer set RAL08, while rows E–H show primer set RAL10. Wells 1 and 49 contain *D. purpurea* ‘Camelot Lavender’ leaf issue, and wells 2 and 50 contain *D. purpurea* ‘Alba’ leaf tissue. Wells 3–6 and 51–54 contain *D. purpurea* ‘Camelot Lavender’ flower, stem, seed pods, and root, and wells 7–10 and 55–58 contain ‘Alba’ flower, stem, seed pods, and root. Wells 11 and 59 are negative controls for the tissue test PCR. Wells 13, 14, 61, and 62 were used to confirm there was no background amplification in 0% biomass *Digitalis* matrix samples. Additionally, 0.5%, 1%, and 5% biomass spike concentrations of *D. lanata* (wells 15–20 and 63–68) and *D. purpurea* ‘Camelot Lavender’ (wells 21–26 and 69–74) were tested in duplicate for both species from lowest to highest. Extraction negatives were run in wells 27, 28, 75, and 76, and PCR negatives in wells 30 and 78. *Digitalis lanata* was used as a positive control in wells 29 and 77. The final row for each primer set was run with a DNA dilution series with 10 ng/µL, 1 ng/µL, 0.1 ng/µL, 0.01 ng/µL, 0.001 ng/µL, and 0.0001 ng/µL from left to right for both *D. purpurea* ‘Camelot Lavender’ (37–42 and 86–90) and *D. lanata* (43–48 and 91–96).

To test the utility of these primers across all tissues of *Digitalis*, roots, stems, leaves, flowers, and seed pods were amplified with RAL08 and RAL10. In all cases except the roots of *D. purpurea* ‘Alba’ tested with RAL08, a dark band was acquired (Figure 3). The RAL10 band for ‘Alba’ was fainter than the others.

### Leaf spikes and assay sensitivity

When extracted using the Qiagen Mericon Food Kit, all samples including the 0% spikes showed bands for the duplicate samples using the universal primer set ITS2 for the positive control (DNA from *D. purpurea* ‘Camelot Lavender’ leaf) but not for the negative controls (Figure 3). When RAL08 and RAL10 were used, the 0% controls were negative for both sets of samples and the 0.5%, 1%, and 5% all had dark bands for both species of *Digitalis* (Figure 3). This experiment showed that *Digitalis* can be detected down to 0.5% biomass by dry weight in both *D. purpurea* and *D. lanata* using these two primer sets in “real life” commercial samples.

As an aside, these samples were initially extracted using the Qiagen Plant DNeasy Mini Kit. However, blending the samples together prior to extraction caused inhibition in the downstream PCR (data not shown) that was clearly visible when the control assay was conducted. A follow‐up dilution series proved that inhibition was the culprit, as the 1:10 and 1:100 dilutions resulted in clear bands. Undilute samples re‐extracted with the Qiagen Mericon Food Kit did not show PCR inhibition.

### Limit of detection

Both *D. purpurea* and *D. lanata* could be detected clearly over three orders of magnitude, or to 0.1 ng, using both primer sets. Additionally, RAL10 could faintly detect down to 0.01 ng for *D. purpurea* (Figure 3).

### Phylogenetic tree generation

The 688‐bp RAL08 barcode trees generated with UPGMA and RAxML showed congruent topography and high support (>75 for UPGMA and 100 for RAxML) for all clades except for the closely related individuals within the *D. purpurea* cultivar clade (Figure 2).

The ITS2 tree created for Figure 1 was intended to visualize the breadth and distribution of samples tested in this study, not to provide phylogenetic inference. However, this tree is congruent with the recently published plastid phylogeny in Xie et al. (2023).

## DISCUSSION

Although there are a multitude of well‐established conserved targets for non‐specific amplification of different organisms (Fazekas et al., 2008; Chen et al., 2010; Handy et al., 2011), identifying and selecting nuclear targets that are taxa specific can be an arduous task. The SISRS‐based primer design method described in this paper provides a blueprint for the development of specific assays for any taxa of interest. SISRS requires an initial WGS dataset from the parent taxa and the target taxa to identify specific SNP‐rich regions, but it is a reference‐free technique that does not require assembled genomes (Schwartz et al., 2015). The number of sites identified scales linearly with the amount of input data (Hunter et al., 2021), and thus the primer design process can be adjusted to any level of implementation, if the appropriate breadth of data is sampled. Here we have modeled the process of development, optimization, and testing for genus‐ and species‐level identification of *Digitalis*, but this process can readily be extended to any taxa of interest.

At the chosen annealing temperatures (Table 3), RAL08 and RAL10 successfully amplified DNA from all five *Digitalis* species tested and did not produce background amplification in any of the 46 Plantaginaceae family members evaluated or in any components of the 13 commercial mixed botanical samples. Amplicons from the longer RAL08 primer set (Table 1) were sequenced to confirm the species‐level differences identified by the primer design process, and each species of *Digitalis* tested had suitable differences to make species‐level determinations if this level of differentiation is desired. The shorter RAL10 amplicons (Table 1) may perform better in highly fragmented samples, such as processed foods, which often yield degraded DNA. While faint, RAL10 was able to amplify the *D. purpurea* ‘Alba’ root tissue, while RAL08 could not, potentially due to DNA fragmentation. Based on these results, when run in tandem, these primers can be used as an additional control to confirm identification of *Digitalis*, but they can also stand alone.

Both primer sets could detect down to 0.5% biomass by weight of two species of *Digitalis*, the lowest level tested. *Digitalis* DNA could be detected to three orders of magnitude (down to 0.1 ng) with both primer sets and to four orders of magnitude (down to 0.01 ng) with RAL10 in *D. purpurea*. These results are concordant with the results obtained in Hunter et al. (2021), where *Digitalis* contamination in the in silico simulated spiked sample data was reliably distinguished from background reads at levels as low as 0.05% biomass. In silico data are useful for initial testing and benchmarking of novel methods, but because these simulations do not perfectly model the artifacts created by the sequencing process or account for differences in laboratory preparation and sample quality, a method's capabilities are often overestimated (Duncavage et al., 2023).

The primer development strategy used here was agnostic to target, and the intention was merely to find a region that would be diagnostic of a genus. However, the RAL08 amplicon appears to contain an exon of a *tRNA‐specific adenosine deaminase 2* (*TAD2‐like*) gene based on BLASTN analysis (Altschul et al., 1990); the RAL10 amplicon is too short to produce a meaningful alignment in BLAST. Although delving into phylogenetic differences was not the focus of this work, especially given the short length of the amplicon, we noted that the RAL08 amplicon for *D. purpurea* ‘Alba’ contained polymorphic bases in four locations that were not present in any other *Digitalis* species sequenced here (Figure 2), which may be a remnant of selective breeding. The taxonomic history of the ‘Alba’ cultivar is convoluted. It was initially classified as its own species, *D. alba* Schrank (Schrank, 1789), but is now considered synonymous with *D. purpurea* (Govaerts et al., 2021). This taxon may be one of the earliest cultivars of the species, having been propagated in the United States for its all‐white flowers by the Custis family in colonial Virginia as early as 1735 (Sale, 1923). Since then, ‘Alba’ has been recognized as having lower glycoside potency (Watanabe, 1923; Cushny, 1925) and concentrations (Ardelean et al., 2006) than more colorful cultivars of *D. purpurea*. Using hybridization studies, Regnart (1935) determined that *D. purpurea* ‘Alba’ is a pure‐breeding, heterotic plant that leads to reproductively sterile offspring when used to produce *Digitalis* hybrids. Our work with the RAL08 primers targeting the nuclear genome showed polymorphic base pair shifts at four locations, possibly indicating inbreeding of this cultivar, as has been demonstrated in cultivated versus wild populations of *D. lutea* (Sandner et al., 2022). This may have also contributed to the fact that when *D. purpurea* ‘Alba’ was used in the gradient test for RAL10 (Appendix S3), it produced dark bands at all temperatures from 50–60°C, whereas cultivar *D. purpurea* ‘Camelot Lavender’ only gave dark bands at 50–56°C.

When assessing food products for composition or contamination, it is important to prove (1) that measurable DNA was extracted from the product of interest and (2) that this DNA is amplifiable and free of inhibitors or fragmentation so severe that it impacts amplification. To this end, it is necessary to use a general primer set (e.g., *rbcL* or ITS2) as a control in conjunction with targeted primer sets. This approach has been successfully used to detect toxic plant contaminants in honey (Wang et al., 2024) and vegetable mixes (Bai et al., 2022). With the commercial samples, ITS2 primers were used as controls to confirm DNA viability, and samples of botanically rich spice mixes spiked with *Digitalis* were subsequently assayed with RAL08‐ and RAL10‐targeted primers to detect contamination. While additional testing to confirm specificity will be valuable, this assay was rigorously assessed across a broad range of confamilial species representing seven out of the 11 remaining tribes of Plantaginaceae, as well as several complex consumer products containing 69 different botanical ingredients. These experiments provide substantial support for the precision and ruggedness of the assay developed here and offer a powerful example of how WGS data can be leveraged to develop targeted tools to protect the food supply.

### Conclusions

The rise in popularity of botanical food products and dietary supplements necessitates rapid, accurate methods to detect potential toxic plant contaminants when adverse events are reported. In cases of emergent outbreaks, such as the 2019 *Datura stramonium* L. contamination of humanitarian relief food (Mutebi et al., 2022), targeted primers could have helped identify the source of the outbreak in considerably less time. This paper presents the development and testing of a targeted qualitative method to detect *Digitalis* contamination in complex food matrices, leveraging WGS data with the SISRS pipeline to identify unique nuclear amplicon sites for *Digitalis* species. The novel method described here for primer development serves as a proof of concept for developing targeted assays from WGS data, and these findings can be used to develop larger, more comprehensive panels specific for botanical species that present a high risk of contamination and toxicity in consumer products, with future goals including other toxic plant taxa such as oleander, jimsonweed, monkshood, and hemlock.

## AUTHOR CONTRIBUTIONS

E.S.H., R.L., and S.M.H. initiated the focus on generation of primers targeting toxic plants using genomic DNA at the U.S. Food and Drug Administration. E.S.H., S.M.H., J.L.W., R.H.U., and S.F. collected, extracted, and processed samples. R.L. performed all computational analyses and generated primer sequences. S.M.H. and S.F. performed laboratory studies with input from E.S.H. E.S.H. and S.M.H. prepared the first draft of this manuscript with input from J.L.W., R.H.U., and R.L. S.M.H. oversaw all aspects of the study. All authors approved the final version of the manuscript.

## Supporting information


**Appendix S1.** Listed ingredients in each of the 13 consumer samples tested, including EM‐27 and EM‐28, which were combined and used as the background for the spiked samples. Note that not all common names refer to a single species, which is why multiple species names are listed in some cases.
**Appendix S2.** GenBank accession numbers for the 334 ITS2 sequences used in the final version of the representative phylogeny shown in Figure 1, as well as 11 different RAL08 barcodes from expertly identified *Digitalis* specimens sequenced as part of this study.

**Appendix S3.** Two gradient gels run on various species of *Digitalis* to test primer amplification of target species across different temperatures. Each primer and species combination tested was run in a vertical column, and the temperature gradient ranged from 60°C (row A) to 50°C (row H). All controls ran as expected.

**Appendix S4.** Temperature gradient showing *Digitalis* species in wells A1–A12 and non‐*Digitalis* Plantaginaceae species in wells B1–E12 at temperatures 50°C (top), 55°C (middle), and 56°C (bottom).

## Data Availability

Whole genome sequencing data used for primer design was sourced from Hunter et al. (2021) and publicly available databases. National Center for Biotechnology Information (NCBI) GenBank accession numbers for Sanger sequence data are provided in Appendix S2.

## References

[aps370013-bib-0001] Albach, D. C. , H. M. Meudt , and B. Oxelman . 2005. Piecing together the “new” Plantaginaceae. American Journal of Botany 92: 297–315. 21652407 10.3732/ajb.92.2.297

[aps370013-bib-0002] Altschul, S. F. , W. Gish , W. Miller , E. W. Myers , and D. J. Lipman . 1990. Basic local alignment search tool. Journal of Molecular Biology 215: 403–410. 2231712 10.1016/S0022-2836(05)80360-2

[aps370013-bib-0003] Ardelean, M. , A. M. Costea , and M. Cordea . 2006. Breeding foxglove (*Digitalis* sp.) for ornamental and/or medical purposes. Symposium on Prospects for the 3rd Millennium Agriculture. Bulletin of University of Agricultural Sciences and Veterinary Medicine 63: 22–31.

[aps370013-bib-0004] Bai, X. , G. Wang , Y. Ren , and J. Han . 2022. Detection of highly poisonous *Nerium oleander* using quantitative real‐time PCR with specific primers. Toxins 14: 776. 10.3390/toxins14110776 36356026 PMC9696062

[aps370013-bib-0005] Beller, G. A. , T. W. Smith , W. H. Abelmann , E. Haber , and W. B. Hood, Jr. 1971. *Digitalis* intoxication: A prospective clinical study with serum level correlations. New England Journal of Medicine 284: 989–997. 5553483 10.1056/NEJM197105062841801

[aps370013-bib-0006] Bohmann, K. , S. Mirarab , V. Bafna , and M. T. P. Gilbert . 2020. Beyond DNA barcoding: The unrealized potential of genome skim data in sample identification. Molecular Ecology 29: 2521–2534. 32542933 10.1111/mec.15507 PMC7496323

[aps370013-bib-0007] Boskabadi, J. , Z. Askari , Z. Zakariaei , M. Fakhar , and R. Tabaripour . 2021. Mild‐to‐severe poisoning due to *Conium maculatum* as toxic herb: A case series. Clinical Case Reports 9: e04509. 34322257 10.1002/ccr3.4509 PMC8299090

[aps370013-bib-0008] Breitwieser, F. P. , J. Lu , and S. L. Salzberg . 2019. A review of methods and databases for metagenomic classification and assembly. Briefings in Bioinformatics 20: 1125–1136. 29028872 10.1093/bib/bbx120 PMC6781581

[aps370013-bib-0009] Bryant, J. P. , F. S. Chapin III , and D. R. Klein. 1983. Carbon/nutrient balance of boreal plants in relation to vertebrate herbivory. Oikos 40: 357–368.

[aps370013-bib-0010] Castello, L. M. , S. Negro , F. Santi , I. Zanotti , M. Vidali , M. Bagnati , G. Bellomo , and G. C. Avanzi . 2012. Accidental digitoxin intoxication: An interplay between laboratory and clinical medicine. Biochemia Medica 22: 380–384. 23092069 10.11613/bm.2012.040 PMC3900044

[aps370013-bib-0011] Chan, S. E. , and C. A. Smith . 2023. A food product as a potential serious cause of liver injury. Clinical Toxicology 61: 616–619. 37706365 10.1080/15563650.2023.2256469

[aps370013-bib-0012] Chen, S. , H. Yao , J. Han , C. Liu , J. Song , L. Shi , Y. Zhu , X. Ma , et al. 2010. Validation of the ITS2 region as a novel DNA barcode for identifying medicinal plant species. PLoS ONE 5: e8613. 20062805 10.1371/journal.pone.0008613 PMC2799520

[aps370013-bib-0013] Chen, F. , W. Dong , J. Zhang , X. Guo , J. Chen , Z. Wang , Z. Lin , et al. 2018. The sequenced angiosperm genomes and genome databases. Frontiers in Plant Science 9: 418. 10.3389/fpls.2018.00418 29706973 PMC5909171

[aps370013-bib-0014] Coutinho Moraes, D. , D. Still , M. Lum , and A. Hirsch . 2015. DNA‐based authentication of botanicals and plant‐derived dietary supplements: Where have we been and where are we going? Planta Medica 81: 687–695. 25856442 10.1055/s-0035-1545843

[aps370013-bib-0015] Cushny, A. R. 1925. The action and uses in medicine of Digitalis and its allies. Longmans, Green and Company, London, United Kingdom.

[aps370013-bib-0016] Duncavage, E. J. , J. F. Coleman , M. E. de Baca , S. Kadri , A. Leon , M. Routbort , S. Roy , et al. 2023. Recommendations for the use of in silico approaches for next‐generation sequencing bioinformatic pipeline validation: A Joint Report of the Association for Molecular Pathology, Association for Pathology Informatics, and College of American Pathologists. Journal of Molecular Diagnostics 25: 3–16. 10.1016/j.jmoldx.2022.09.007 36244574

[aps370013-bib-0017] Fazekas, A. J. , K. S. Burgess , P. R. Kesanakurti , S. W. Graham , S. G. Newmaster , B. C. Husband , D. M. Percy , et al. 2008. Multiple multilocus DNA barcodes from the plastid genome discriminate plant species equally well. PLoS ONE 3: e2802. 18665273 10.1371/journal.pone.0002802 PMC2475660

[aps370013-bib-0018] Fu, L. , B. Niu , Z. Zhu , S. Wu , and W. Li . 2012. CD‐HIT: Accelerated for clustering the next‐generation sequencing data. Bioinformatics 28: 3150–3152. 23060610 10.1093/bioinformatics/bts565 PMC3516142

[aps370013-bib-0019] Gafner, S. , M. Blumenthal , S. Foster , J. H. Cardellina , I. A. Khan , and R. Upton . 2023. Botanical ingredient forensics: Detection of attempts to deceive commonly used analytical methods for authenticating herbal dietary and food ingredients and supplements. Journal of Natural Products 86: 460‐472. 36716213 10.1021/acs.jnatprod.2c00929 PMC9972475

[aps370013-bib-0020] Govaerts, R. , E. Nic‐Lughadha , N. Black , R. Turner , and A. Paton . 2021. The world checklist of vascular plants, a continuously updated resource for exploring global plant diversity. Scientific Data 8: 215. 10.1038/s41597-021-00997-6. 34389730 PMC8363670

[aps370013-bib-0021] Handy, S. M. , J. R. Deeds , N. V. Ivanova , P. D. Hebert , R. H. Hanner , A. Ormos , L. A. Weigt , et al. 2011. A single‐laboratory validated method for the generation of DNA barcodes for the identification of fish for regulatory compliance. Journal of AOAC International 94: 201–210. 21391497

[aps370013-bib-0022] Hunter, E. S. , R. Literman , and S. M. Handy . 2021. Utilizing big data to identify tiny toxic components: *Digitalis* . Foods 10: 1794. 34441571 10.3390/foods10081794 PMC8391216

[aps370013-bib-0023] Ivanova, N. V. , M. L. Kuzmina , T. W. Braukmann , A. V. Borisenko , and E. V. Zakharov . 2016. Authentication of herbal supplements using next‐generation sequencing. PLoS ONE 11: p.e0156426. 27227830 10.1371/journal.pone.0156426 PMC4882080

[aps370013-bib-0024] Karthikeyan, A. , and S. D. Amalnath . 2017. *Abrus precatorius* poisoning: A retrospective study of 112 patients. Indian Journal of Critical Care Medicine 21: 224–225. 28515607 10.4103/ijccm.IJCCM_320_16 PMC5416790

[aps370013-bib-0025] Katoh, K. , and D. M. Standley . 2013. MAFFT Multiple Sequence Alignment Software Version 7: Improvements in performance and usability. Molecular Biology and Evolution 30: 772–780. 23329690 10.1093/molbev/mst010 PMC3603318

[aps370013-bib-0026] Khilare, V. , A. Tiknaik , B. Prakash , B. Ughade , G. Korhale , D. Nalage , N. Ahmed , et al. 2019. Multiple tests on saffron find new adulterant materials and reveal that 1st grade saffron is rare in the market. Food Chemistry 272: 635–642. 30309592 10.1016/j.foodchem.2018.08.089

[aps370013-bib-0027] Kocyigit, E. , B. Kocaadam‐Bozkurt , O. Bozkurt , D. Ağagündüz , and R. Capasso . 2023. Plant toxic proteins: Their biological activities, mechanism of action and removal strategies. Toxins 15: 356. 10.3390/toxins15060356 37368657 PMC10303728

[aps370013-bib-0028] Kreis, W. 2017. The foxgloves (*Digitalis*) revisited. Planta Medica 83(12/13): 962–976. 28561136 10.1055/s-0043-111240

[aps370013-bib-0029] Lapostolle, F. , and S. W. Borron . 2007. Digitalis. *In* M. W. Shannon , S. W. Borron , and M. J. Burns [eds.], Haddad and Winchester's clinical management of poisoning and drug overdose, 4th ed., 949–962. Saunders, Philadelphia, Pennsylvania, USA.

[aps370013-bib-0030] Li, X. , Y. Yang , R. J. Henry , M. Rossetto , Y. Wang , and S. Chen . 2015. Plant DNA barcoding: From gene to genome: Plant identification using DNA barcodes. Biological Reviews 90: 157–166. 24666563 10.1111/brv.12104

[aps370013-bib-0031] Lin, C. C. , C. C. Yang , D. H. Phua , J. F. Deng , and L. H. Lu . 2010. An outbreak of foxglove leaf poisoning. Journal of the Chinese Medical Association 73: 97–100. 20171590 10.1016/S1726-4901(10)70009-5

[aps370013-bib-0032] Maffè, S. , L. Cucchi , F. Zenone , C. Bertoncelli , F. Beldì , M. L. Colombo , M. Bielli , et al. 2009. *Digitalis* must be banished from the table: A rare case of acute accidental *Digitalis* intoxication of a whole family. Journal of Cardiovascular Medicine 10: 727–732. 19491701 10.2459/JCM.0b013e32832c2314

[aps370013-bib-0033] Mutebi, R. R. , A. R. Ario , M. Nabatanzi , I. B. Kyamwine , Y. Wibabara , P. Muwereza , D. Eurien , et al. 2022. Large outbreak of Jimsonweed (*Datura stramonium*) poisoning due to consumption of contaminated humanitarian relief food: Uganda, March–April 2019. BMC Public Health 22: 623. 35354446 10.1186/s12889-022-12854-1 PMC8969350

[aps370013-bib-0034] Negroni, M. S. , A. Marengo , D. Caruso , A. Tayar , P. Rubiolo , F. Giavarini , S. Persampieri , et al. 2019. A case report of accidental intoxication following ingestion of foxglove confused with borage: High digoxinemia without major complications. Case Reports in Cardiology 2019: 9707428. 31871798 10.1155/2019/9707428 PMC6906804

[aps370013-bib-0035] Pearman, W. S. , N. E. Freed , and O. K. Silander . 2020. Testing the advantages and disadvantages of short‐ and long‐read eukaryotic metagenomics using simulated reads. BMC Bioinformatics 21: 220. 32471343 10.1186/s12859-020-3528-4 PMC7257156

[aps370013-bib-0036] Pincus, M. 2016. Management of digoxin toxicity. Australian Prescriber 39: 18–20. 27041802 10.18773/austprescr.2016.006 PMC4816869

[aps370013-bib-0037] Price, M. N. , P. S. Dehal , and A. P. Arkin . 2010. FastTree 2 – Approximately maximum‐likelihood trees for large alignments. PLoS ONE 5: e9490. 20224823 10.1371/journal.pone.0009490 PMC2835736

[aps370013-bib-0038] Quinlan, A. R. , and I. M. Hall . 2010. BEDTools: A flexible suite of utilities for comparing genomic features. Bioinformatics 26: 841–842. 10.1093/bioinformatics/btq033 20110278 PMC2832824

[aps370013-bib-0039] R Core Team . 2024. R: A language and environment for statistical computing. R Foundation for Statistical Computing, Vienna, Austria. Website https://www.R-project.org/ [accessed 9 May 2024].

[aps370013-bib-0040] Regnart, H. C. 1935. Studies of hybrids in the genus *Digitalis*: I. The cytology of a sterile hybrid between *Digitalis dubia* and *Digitalis purpurea* . Genetica 17: 145–153.

[aps370013-bib-0041] Sale, E. D. T. 1923. Historic gardens of Virginia. The James River Garden Club, Richmond, Virginia, USA.

[aps370013-bib-0042] San Andrés Larrea, M. I. , M. D. San Andrés Larrea , and L. Alcides Olivos‐Oré. 2024. Plants, Poisonous (Animals). *In* Philip Wexler [ed.], Encyclopedia of toxicology, 4th ed., 685–703. Academic Press, Cambridge, Massachusetts, USA.

[aps370013-bib-0043] Sandner, T. M. , B. Gemeinholzer , J. Lemmer , D. Matthies , and A. Ensslin . 2022. Continuous inbreeding affects genetic variation, phenology, and reproductive strategy in *ex situ* cultivated *Digitalis lutea* . American Journal of Botany 109: 1545–1559. 36164840 10.1002/ajb2.16075

[aps370013-bib-0044] Schrank, F. V. P. 1789. Bayerische Flora, Erster Band. J. B. Strobl, Munich.

[aps370013-bib-0045] Schwartz, R. S. , K. M. Harkins , A. C. Stone , and R. A. Cartwright . 2015. A composite genome approach to identify phylogenetically informative data from next‐generation sequencing. BMC Bioinformatics 16: 193. 26062548 10.1186/s12859-015-0632-y PMC4464851

[aps370013-bib-0046] Slifman, N. R. , W. R. Obermeyer , B. K. Aloi , S. M. Musser , W. A. Correll, Jr. , S. M. Cichowicz , J. M. Betz , and L. A. Love . 1998. Contamination of botanical dietary supplements by *Digitalis lanata* . New England Journal of Medicine 339: 806–811. 9738088 10.1056/NEJM199809173391204

[aps370013-bib-0047] Stamatakis, A. 2014. RAxML version 8: A tool for phylogenetic analysis and post‐analysis of large phylogenies. Bioinformatics 30: 1312–1313. 24451623 10.1093/bioinformatics/btu033 PMC3998144

[aps370013-bib-0048] Walker, K. M. , and W. L. Applequist . 2012. Adulteration of selected unprocessed botanicals in the US retail herbal trade. Economic Botany 66: 321–327.

[aps370013-bib-0049] Wang, G. , Y. Ren , Y. Su , H. Zhang , J. Li , H. Zhao , H. Zhang , and J. Han . 2024. Identification of toxic *Gelsemium elegans* in processed food and honey based on real‐time PCR analysis. Food Research International 182: 114188. 10.1016/j.foodres.2024.114188 38519193

[aps370013-bib-0050] Watanabe, M. 1923. On the pharmacological efficacy of *Digitalis* leaf. The Tohoku Journal of Experimental Medicine 4: 98–148.

[aps370013-bib-0051] Wickham, H. 2016. ggplot2: Elegant graphics for data analysis. Springer‐Verlag, New York, New York, USA.

[aps370013-bib-0052] Worbs, S. , K. Köhler , D. Pauly , M. A. Avondet , M. Schaer , M. B. Dorner , and B. G. Dorner . 2011. *Ricinus communis* intoxications in human and veterinary medicine—A summary of real cases. Toxins 3: 1332–1372. 22069699 10.3390/toxins3101332 PMC3210461

[aps370013-bib-0053] World Flora Online . 2024. World Flora Online database. Website http://www.worldfloraonline.org/ [accessed 21 May 2025].

[aps370013-bib-0054] Xie, P. , L. Tang , Y. Luo , C. Liu , and H. Yan . 2023. Plastid phylogenomic insights into the inter‐tribal relationships of Plantaginaceae. Biology 12: 263. 36829541 10.3390/biology12020263 PMC9953724

[aps370013-bib-0055] Zhang, N. , P. Ramachandran , J. Wen , J. A. Duke , H. Metzman , W. McLaughlin , A. R. Ottesen , et al. 2017. Development of a reference standard library of chloroplast genome sequences, GenomeTrakrCP. Planta Medica 83: 1420–1430. 28651291 10.1055/s-0043-113449

