# Supplementary material for: From WGS to gels: Development and testing of PCR primers targeting toxic Digitalis in support of food safety
Source: Appl Plant Sci. 2025 Jul 1;13(5):e70013. doi: 10.1002/aps3.70013 (PMC12542805; doi:10.1002/aps3.70013)

DOI 10.1002/aps3.70013

APPENDIX S3. Two gradient gels run on various species of *Digitalis* to test primer amplification of target species across different temperatures. Each primer and species combination tested was run in a vertical column, and the temperature gradient ranged from 60°C (row A) to 50°C (row H). All controls ran as expected.

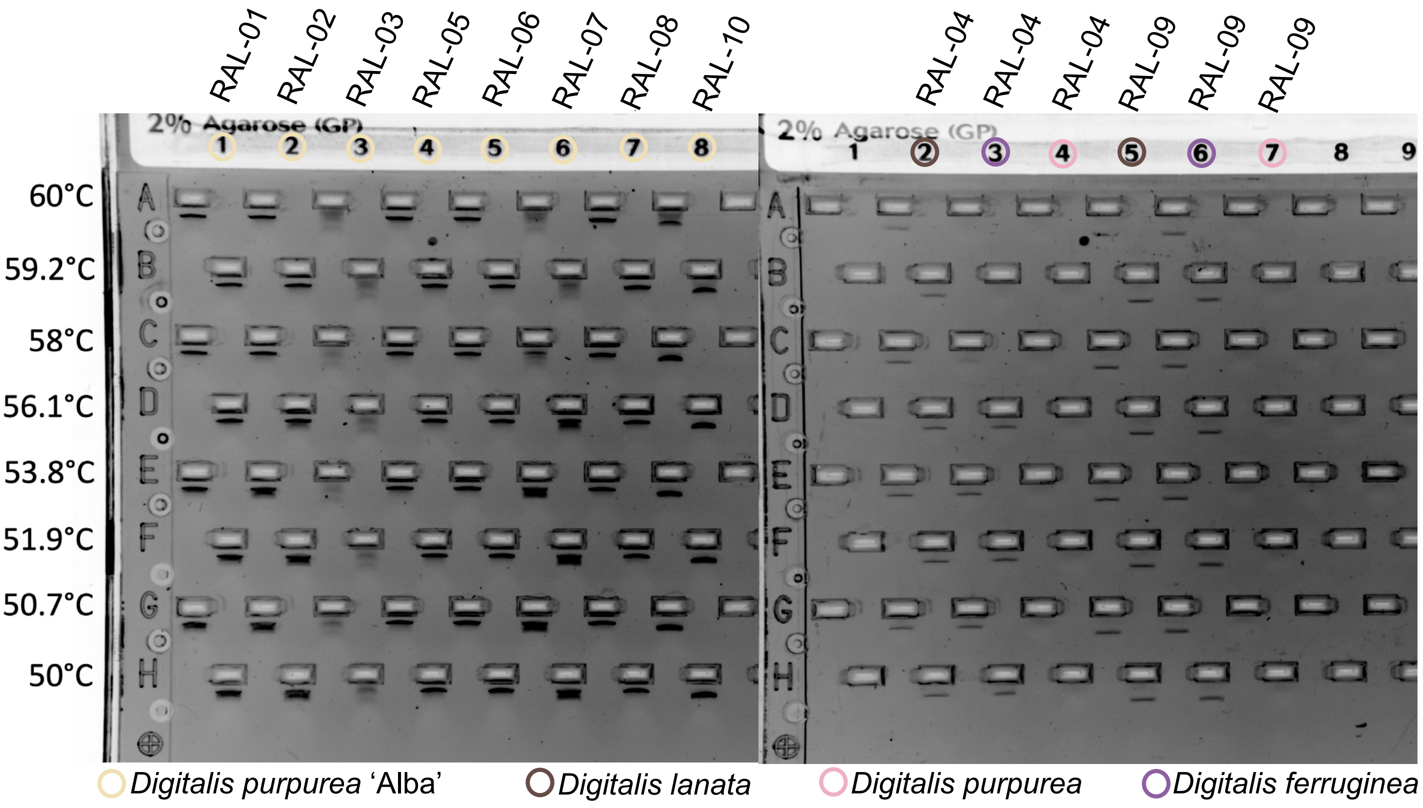

Supplement: Supplementary file 2 — Appendix S3. Two gradient gels run on various species of Digitalis to test primer amplification of target species across different temperatures. Each primer and species combination tested was run in a vertical column, and the temperature gradient ranged from 60°C (row A) to 50°C (row H). All controls ran as expected. [file APS3-13-e70013-s002.pdf]
