# Supplementary material for: From WGS to gels: Development and testing of PCR primers targeting toxic Digitalis in support of food safety
Source: Appl Plant Sci. 2025 Jul 1;13(5):e70013. doi: 10.1002/aps3.70013 (PMC12542805; doi:10.1002/aps3.70013)

DOI 10.1002/aps3.70013

APPENDIX S4. Temperature gradient showing *Digitalis* species in wells A1–A12 and non-*Digitalis* Plantaginaceae species in wells B1–E12 at temperatures 50°C (top), 55°C (middle), and 56°C (bottom).

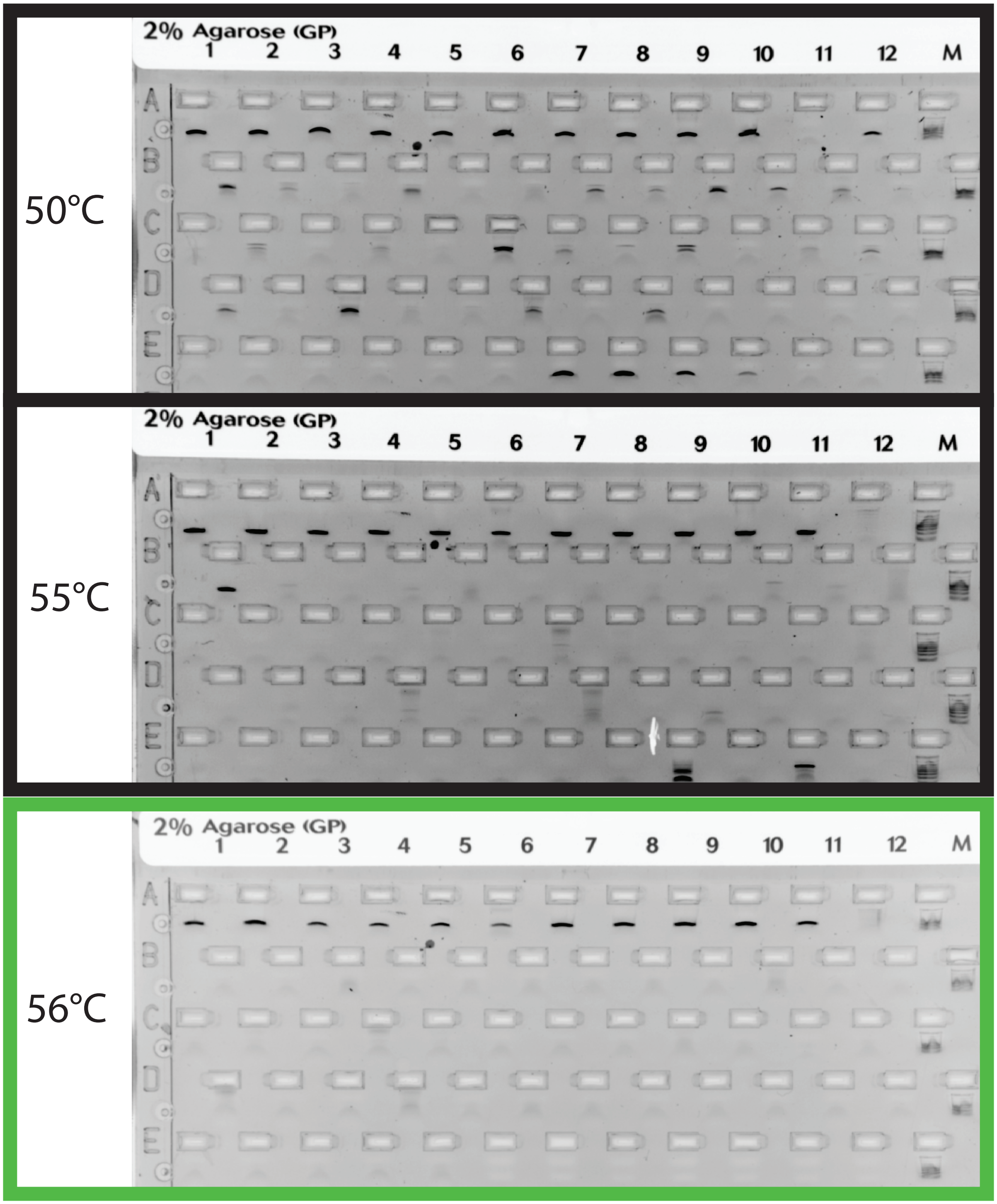

Supplement: Supplementary file 3 — Appendix S4. Temperature gradient showing Digitalis species in wells A1–A12 and non‐Digitalis Plantaginaceae species in wells B1–E12 at temperatures 50°C (top), 55°C (middle), and 56°C (bottom). [file APS3-13-e70013-s001.pdf]
